# Detection of clinically relevant antibiotic-resistant bacteria in shared fomites, waste water and municipal solid wastes disposed near residential areas of a Nigerian city

**DOI:** 10.1099/acmi.0.000641.v4

**Published:** 2023-12-15

**Authors:** Ibrahim Yusuf, Zainab Damji Muhammad, Binta Muhammad Amin, Maryam Danladi Shuaibu, Nafisatu Hamza, Hajara Dauda Isah, Nasir Bako Abdullahi, Patience James Ene, Sameera Salisu Shuaibu, Nasir Doguwa, Shamsudeen Lekan Pedro, Maryam Adamu Muhammad

**Affiliations:** ^1^​ Department of Microbiology, Faculty of Life Sciences, College of Natural and Pharmaceutical Sciences, Bayero University, Kano, P.M.B. 3011, Kano, Nigeria; ^2^​ Department of Microbiology, Aminu Kano Teaching Hospital, Kano, Nigeria; ^3^​ Center for Biotechnology Research, Bayero University, Kano, Niger

**Keywords:** antibiotic-resistant bacteria, antibiotic resistance genes, environmental waste, fomites, municipal solid wastes, Nigeria, restroom floors, sewage, sludge, waste water

## Abstract

Studies investigating environmental hotspots of antibiotic-resistant bacteria (ARB) and antibiotic resistance genes (ARGs) in Nigeria are limited. This study was designed to assess various environmental sources and commonly touched surfaces as potential carriers of ARB and ARGs with implications for public health. A total of 392 samples, including sewage (36), sludge (36), diapers (20), plastics (20), water sachet polythene bags (20), food wastes (20), soil beneath dump sites (20), and frequently touched surfaces such as restroom floors (80), corridors (24), door handles (56), and room floors and walls (60), were collected and screened for the presence of resistant bacteria carrying genes such as *bla*_*KPC*_, *bla*_*NDM-1*_, *bla*_*CMY-2*_, *bla*_*IMP*_, *bla*_*OXA66*_ and *MecA*. Additionally, we employed standard techniques to detect methicillin-resistant *Staphylococcus aureus* (MRSA) and extended-spectrum β-lactamase (ESBL)-producing *Escherichia coli, Klebsiella pneumoniae, Pseudomonas aeruginosa* and *Acinetobacter baumannii *. We also evaluated the effectiveness of routine disinfection procedures in eliminating ARB from restroom floors. Our findings revealed that sewage, sludge, diapers, food wastes and restroom floors are frequently contaminated with highly and moderately resistant strains of *E. coli, K. pneumoniae, P. aeruginosa* and MRSA. Notably, we identified two variants of the *bla*_*OXA51-like*_ gene (*bla*_*OXA-66*_ and *bla*_*OXA-180*_) in *A. baumannii* isolated from these environmental sources. Furthermore, we detected seven ESBL-* K. pneumoniae *, five ESBL-* A. baumannii *, two ESBL-* E. coli* and one ESBL-* P. aeruginosa *, all carrying one or more ARGs (*bla*_*KPC*_, *bla*_*NDM-1*_, *bla*_*CMY-2*_), in isolates recovered from sewage, sludge, restroom floors and plastics. It is of note that ARB persisted on restroom floors even after disinfection procedures. In conclusion, this study highlights that environmental wastes indiscriminately discarded in residential areas and shared surfaces among individuals are heavily colonized by ARB carrying ARGs of significant public health importance.

## Data Summary

Data generated in this study include bacteria and genomic data. The bacterial isolates are freely available at the Bayero University collection centre on request. Similarly, the genomic datasets are also freely available in GenBank with the following genome accession numbers; ***Acinetobacter baumannii* complete genome depicting different resistant genes**: JAOBQT000000000 (JAOBQT010000001-JAOBQT010000055), JAOBQV000000000 (JAOBQV010000001-JAOBQV010000103), JAOBQS000000000 (JAOBQS010000001- JAOBQS010000069). **Other Gram-negative bacteria ARGs**: OR622914, OR800940, OR800941, OR800942 and OR800943.

## Introduction

The open dumping of municipal solid waste (MSW), without segregation and treatment, is a significant concern due to its hazardous contents of antibiotic-resistant bacteria (ARB), antibiotic resistance genes (ARGs), and other antibiotic resistance-driving agents (ARDAs). Increased interest in determining the role of the environment in driving and spreading ARB and ARGs has led to a renewed interest in surveying different environmental wastes and frequently touched fomites for carrying ARB and ARGs of public health concern.

The overuse of antibiotics in humans and animals has led to continual release of antibiotics through excretory products into terrestrial (e.g. dumpsites) and aquatic (e.g. stagnated waters) ecosystems, which has resulted in the evolution of new ARB and ARGs of great concern to public health [1, 2]. As MSWs are not properly disposed of in most Nigerian cities, the proximity of illegal disposal sites to residential areas makes it easy for microbial populations that have accumulated in the wastes to cross-contaminate humans, animals and shared fomites. In addition to MSW, wastewater and its derivatives (sewage from sewers and sludge from gutters) are also discharged into the environment from sources, such as laundries, kitchens, agricultural land (in the form of runoff ) and industries (in the form of effluents) and are often contaminated with different bacteria, and unquantifiable amounts of ARDAs such as residual antibiotics, disinfectants, metals and other toxic chemicals [3, 4].

In Nigeria, while sewage and sludge frequently stagnate in drainage systems in the form of gutters, MSWs are regularly made up of plastics, water sachet polythene bags, diapers, food wastes, metals, etc., and end up in open areas and drainages.

The presence of high densities of active ARB and ARGs in MSW and fomites could serve as hotspots from where additional ARB could evolve through horizontal transfer of ARGs to other bacteria or place humans and animals scavenging or living in surrounding areas at risk of acquiring and disseminating ARB and ARGs through direct contact. [5, 6]

Despite the worldwide distribution and endemicity of methicillin-resistant *Staphylococcus aureus* (MRSA) and extended spectrum beta-lactamase (ESBL)-producing *Escherichia coli* (ESBL-* E. coli *), *Klebsiella pneumoniae* (ESBL-* K. pneumoniae *), *Pseudomonas aerugiosa* (ESBL-* P. aeruginosa *) and *Acinetobacter baumannii* (ESBL-* A. baumannii *) in clinical settings [7–9], little is known regarding the level of colonization of the major components of MSW, waste water derivatives (WWDs) and fomites with ARB in Nigeria and whether they also harbour ARGs of clinical importance. Previous isolation of carbapenem-resistant *Enterobacteriaceae* in rural communities with no prior use of carbapenem in Nigeria may suggest an environmental carriage and reservoirs which can be transferred to hospital environments via sick or asymptomatic carriers. [9]

A better understanding of burden, ecological niches and reservoirs of MRSA and ESBL-producing bacteria in indiscriminately discharged environmental wastes and shared fomites would help in managing and controlling their spread in hospitals and communities. The objectives of the present study were: (i) to screen major components of MSW, WWDs and shared fomites in a Nigerian city for ARB carriage; (ii) to screen the ARB isolated for carriage of some ARGs of public health concern; and (iii) to evaluate the efficacy of restroom floor disinfection procedures routinely used in the university in reducing the burden of bacterial contaminants.

## Methods

### Study setting and sample collection

Prior to the start of the study, an ethical clearance was sought and provided by the ethical committee of Kano State Ministry of Health. The study was conducted in the Department of Microbiology, Bayero University Kano (BUK), Center for Biotechnology Research (CBR), BUK and Microbiology Department of Aminu Kano Teaching Hospital Kano-Nigeria. A total of 392 samples/swabs were collected between March and June 2021 as follows.

### Solid waste and waste water sampling

**Fig. 1.**
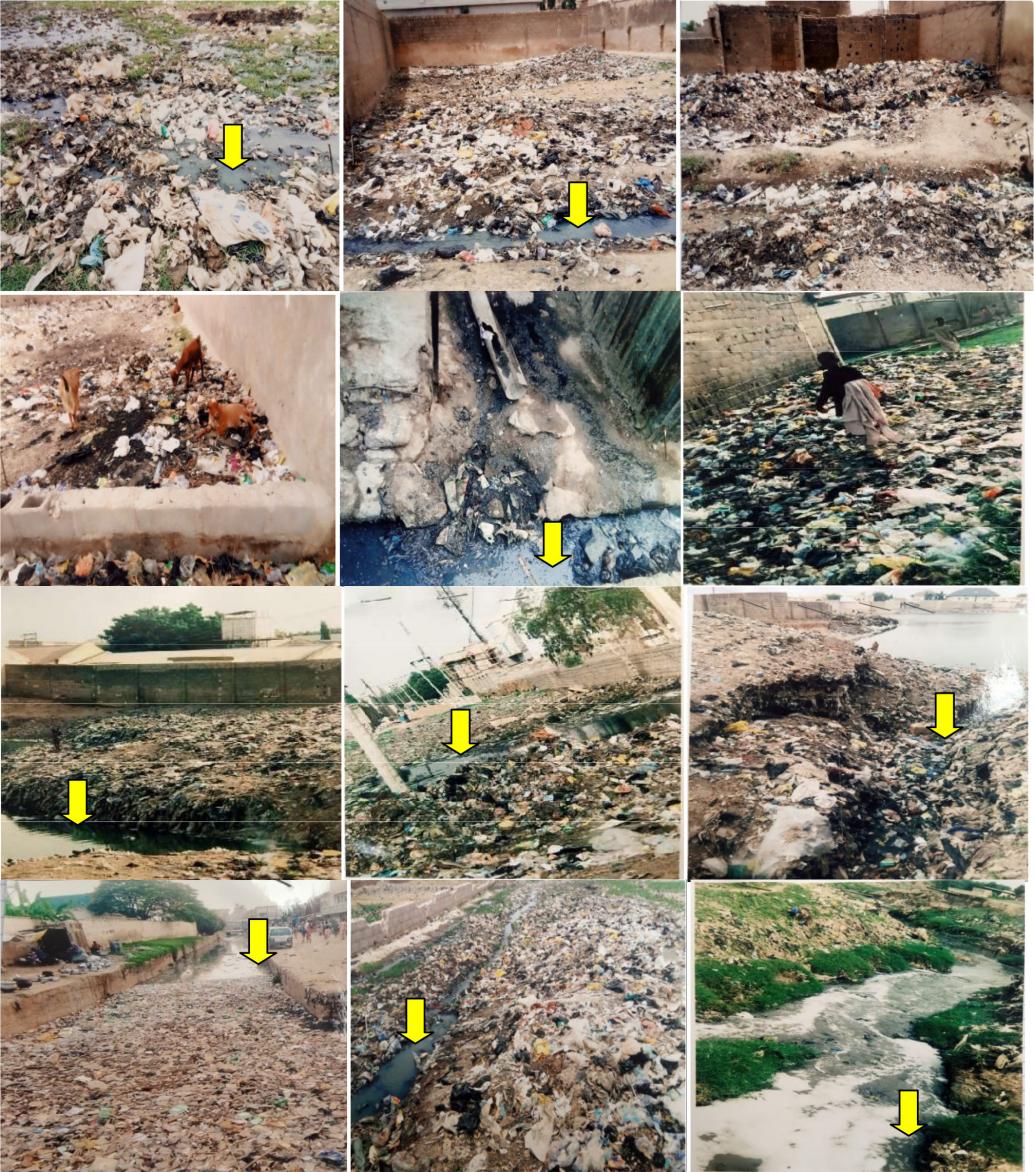
Locations where samples of MSW and waste water were collected. The sampling points were situated near residential areas to which humans (passersby and scavengers) and animals have free access. Each component of MSW was picked from wastes deposited for more than 1 week. Yellow arrows indicate sites where waste water derivatives were sampled (sewage and sludge).

Samples of plastics, diapers, sachet water polythene bags, food wastes and soil from different MSW illegal disposal sites situated around residential areas were collected using sterile forceps and a spatula at random and placed into sterile glass plastic containers. Meanwhile, samples of sewage and sludge were also collected from gutters and open sewers around residential areas at Dorayi Karama, Hotoro Tsamiyar Boka and hostels for men of Bayero University Kano, Nigeria. Sites of sampling and the nature of discharged wastes are shown in Fig. 1. Samples were placed into sterile containers and were transported immediately to the laboratory for processing. In the laboratory, three different surfaces of plastics, diapers and sachet water polythene bags were swabbed to form a composite sampling using a sterile cotton-tipped swab stick.

### Fomite sampling

A 4 day longitudinal sampling of fomites in Nigerian University hostel was undertaken. Samples were collected from two categories of surfaces.

(1) *Restroom floor*: samples were collected across two blocks of hostels comprising five restrooms per block. Restroom floors were sampled with moist swabs from two locations (floors around the toilet seat and floors around the door) in the morning (8 a.m.) before daily cleaning and 1 h after routine cleaning. In addition, samples from the floor of a corridor between the restroom and rooms were also collected.

(2) S*hared rooms*: walls, floors and door handles of rooms shared by students were also sampled using similar techniques.

### Isolation, identification and screening of MRSA and ESBL-producing bacteria

Sewage and sewage samples were appropriately diluted before inoculation onto nutrient agar plates, which were subsequently incubated at 37 °C for 24 h. Following incubation, suspected isolates of *S. aureus, K. pneumoniae, P. aeruginosa* and *E. coli* were sub-cultured onto mannitol salt agar, EMB and MacConkey agar plates.

For each isolate, observations were made regarding its colonial characteristics, including size, shape, colour, margin, elevation and opacity. Additionally, morphological characteristics such as Gram reaction, cell shape and arrangement were determined according to established methods. The identity of the isolates was further confirmed using the API 20E kit (bioMérieux).

Confirmation of *A. baumannii* relied on the detection of *bla*_OXA-51-like_ genes in genomic DNA obtained through the boiling method. Antibiotic susceptibility patterns of all the isolates were determined following the standards specified by the Clinical Laboratory Standards Institute (CLSI). In brief, approximately 150 µl of standardized nutrient broth for each bacterium was streaked onto Mueller–Hinton agar plates (Oxoid) in duplicate. Sixteen antibiotic discs (all from Oxoid) were placed on each plate, including amoxicillin, ampicillin, ceftriaxone, cefoxitin, cefotaxime, cefepime, imipenem, ciprofloxacin, levofloxacin, gentamicin, kanamycin, tetracycline, tigecycline, clindamycin, erythromycin and colistin. After 24 h of incubation at 37 °C under ambient conditions, the diameters of inhibition zones were measured and interpreted according to species-specific CLSI 2018 breakpoints [10].

Furthermore, *S. aureus* isolates were screened for MRSA phenotypically using cefoxitin susceptibility and genotypically by amplification of *mecA* genes. Resistant isolates of *E. coli, P. aeruginosa, K. pneumoniae* and *A. baumannii* were screened for ESBL production using the double disc synergy test, as described in CLSI 2018 [11].

### Detection of ARGs

The ARGs considered in this study include *bla*_KPC_, *bla*_NDM_, *bla*_IMP_ and *bla*_CMY-2_, which confer resistance to commonly used β-lactam and carbapenem antibiotics in the study area. Plasmids were extracted from resistant *E. coli, K. pneumoniae, P. aeruginosa* and *A. baumannii* using a commercial kit (Zymo Research). Subsequently, the selected ARGs were amplified using conventional PCR with their respective oligonucleotides (Table 1). For plasmid DNA extracted from *E. coli, K. pneumoniae* and *P. aeruginosa *, PCR amplification targeted fragments of *bla*_*NDM*_, *bla*_*IMP*_, *bla*_*KPC*_ and *bla*_*CMY-2*_, measuring 476, 587, 882 and 106 bp, respectively. Each PCR consisted of 2 µl of template DNA, 1 µl of primers (at final concentrations of 0.66 µM each) and 12.5 µl of X2 Dream Taq Master Mix (Thermo Scientific), in a final volume of 25 µl. The PCRs underwent 30 cycles using previously described cycling conditions [12]. PCR amplicons were visualized on a 1.5 % agarose gel stained with ethidium bromide for 42 min (150 V, 400 A), and the gel image was captured using a documentation device (OmniDOC Gel Documentation System; Cleaver Scientific). Samples displaying a band were selected, purified using a DNA and PCR purification kit (GDSBios) and subsequently sequenced.

**Table 1.** Genes amplified in resistant Gram-negative bacteria isolated from environmental wastes and fomites

Primers	Sequence	Amplicon size (bp)	Reference
*bla_NDM_*	Forward: CTGAGCACCGCATTAGCC Reverse: GGGCCGTATGAGTGATTGC	476	[12]
*bla_IMP_*	Forward: GAAGGCGTTTATGTTCATAC Reverse: GTATGTTTCAAGAGTGATGC	587	[37]
*bla_KPC_*	Forward: ATGTCACTGTATCGCCGTCT Reverse: TTACTGCCCGTTGACGCCC	882	[38]
*bla_CMY-2_*	Forward: CCGAAGCCTATGCGTGAAATCC Reverse: GCAATGCCCTGCTGGAGCG	106	Designed
*bla_OXA51-like_*	Forward: TAATGCTTTGATCGGCCTTG Reverse: TGGATTGCACTTCATCTTGG	353	[39]
*mecA*	Forward: TCCAGATTACAACTTCACCAGG Reverse: CCACTTCATATCTTGTAACG	162	[40]
*bla_OXA69_*	Oxa69A: CTAATAATTGATCTACTCAAG Oxa69B: CCAGTGGATGGATGGATAGATTATC	801	[41]

For sequencing, 5 µl of purified amplicon was subjected to Sanger sequencing on a 3730xl DNA Analyzer (ThermoFisher Scientific) at Inqaba Biotech. Sequence chromatograms were visually inspected for quality using FinchTv (version 1.4). These sequences were then compared to existing sequences in GenBank (https://www.ncbi.nlm.nih.gov/genbank/) using blast with default parameters to search in the ‘nucleotide collection (nr/nt)’ database (GenBank) for highly similar sequences (megablast) (TBLASTX, RRID:SCR_011823).

To determine the specific *bla*_*OXA-51-like*_ gene variant of the *A. baumannii* isolates, the coding region of the gene was amplified with *bla*_*OXA69*_ primers and fully sequenced according to method described by Pfeifer *et al*. [12] and sequence assembly was performed *de novo* using the Geneious bioinformatic tool for contig assembly. The consensus sequence was extracted, copied and blasted using the translated nucleotides to protein tool (Blastx).

### Efficacy of restroom floor disinfection process in reducing ARB

Because the restroom floors are often disinfected every day, the efficacy of the type of disinfectants used in removing or reducing bacteria or ARB were tested by checking for the presence of ARB and other bacteria 1 h after the cleaning process [23].

### Data analysis

The results are presented in frequency distribution tables and figures. Statistical analysis was performed using the SPSS (RRID:SCR_002865) statistical package (SPSS). A chi-square test was used to compare differences between groups. Statistical significance was set at *P*<0.05.

## Results

### Isolation of bacteria from environmental wastes and shared fomites

Out of the total 392 samples that were screened, 191 (48.7 %) produced positive cultures of *S. aureus *, *E. coli, P. aeruginosa, K. pneumoniae* and *A. baumannii,* each at varying rates. The counts of bacteria for each sample type are given in Table 2. Among all the sampled areas, including MSW, WWD and fomites, the highest bacterial recovery rates were observed in sewage (88.9 %), the sand where samples were disposed of (85 %), sludge (75 %) and restroom floors (37 %). Furthermore, *A. baumannii* was more frequently recovered in WWD, with rates of 11.1 % in sewage and 8.3 % in sludge, as well as in fomites, with rates of 12 % on door handles and 10 % on restroom floors, compared to MSW, where it was only isolated in the sand sample (5 %).

**Table 2.** Overall recovery of bacteria from MSW, WWD and SF

Sample/waste main type	Sample sub-type	No. of samples screened	No. of isolates recovered (%)					Total
			* E. coli *	* S. aureus *	* K. pneumoniae *	* P. aeruginosa *	*A*. *baumannii*	
Waste water derivatives	Sewage	36	7 (21.9)	9 (28.1)	8 (25.0)	4 (12.5)	4 (12.5)	32 (88.8)
	Sludge	36	8 (29.6)	6 (22.2)	9 (33.3)	1 (3.70)	3 (11.1)	27 (75.0)
Municipal solid wastes	Diapers	20	2 (28.6)	2 (28.6)	3 (42.8)	0 (0.0)	0 (0.0)	7 (35.0)
	Plastics	20	3 (23.0)	4 (30.8)	4 (30.8)	2 (15.4)	0 (0.0)	13 (65.0)
	Water sachet polythene bags	20	2 (20.0)	1 (10.0)	4 (40.0)	3 (30.0)	0 (0.0)	10 (35.0)
	Food remains	20	1 (11.1)	3 (33.3)	5 (55.6)	0 (0.0)	0 (0.0)	9 (45.0)
	Sand	20	2 (11.8)	7 (41.2)	4 (23.5)	3 (17.6)	1 (5.9)	17 (85.0)
Shared fomites	Restroom floors	80	9 (24.3)	10 (27.0)	9 (24.3)	1 (2.7)	8 (21.6)	37 (46.2)
	Corridor	24	2 (25.0)	3 (37.5)	3 (37.5)	0 (0.0)	0 (0.0)	8 (33.3)
	Room door handle	56	2 (10.5)	10 (52.6)	3 (15.8)	0 (0.0)	4 (21.1)	19 (33.9)
	Room walls and floors	60	1 (8.3)	6 (50.0)	3 (25.0)	2 (16.7)	0 (0.0)	12 (20.0)
Total		392	39 (20.4)	61 (31.9)	55 (28.8)	16 (8.4)	20 (10.5)	191 (48.7)

### Antibacterial susceptibility of isolated bacteria to antibiotics

Antibiotic sensitivity testing conducted on 39 *E. coli *, 55 *K *. *pneumoniae *, 52 *S *. *aureus *, 16 *P *. *aeruginosa* and 20 *A *. *baumannii* samples revealed that they exhibited high suscebtibiity (90–100 %) to cephalosporins and the carbapenem (imipenem). In contrast, the majority of these isolates demonstrated resistance to ampicillin and amoxicillin, and showed intermediate resistance to fluoroquinolones. Notably, all *A. baumannii* strains exhibited complete resistance to penicillins (ampicillin and amoxicillin), tetracycline, tigecycline, erythromycin, gentamicin, kanamycin and chloramphenicol, but remained susceptible to colistin and imipenem. Approximately 50 % of *P. aeruginosa* strains displayed resistance to ciprofloxacin, levofloxacin and erythromycin (Table 3).

**Table 3.** Percentage susceptibility of bacteria isolated from different MSW, waste water derivatives and shared fomites to multiple antibiotics

Antibiotic class	Antibiotic	* E. coli* (*n*=39)			* K. pneumoniae* (*n*=55)				* S. aureus* (52)			* P. aeruginosa* (16)			* A. baumannii* (20)		
		WWD	MSW	SF	WWD	MSW		SF	WWD	MSW	SF	WWD	MSW	SF	WWD	MSW	SF
Penicillin	Amoxicillin	32	38	43	32		13	32	56	22	51	19	12	12	0	0	0
	Ampicillin	49	27	32	42		32	42	45	31	22	12	31	31	0	0	0
Cephalosporin	Ceftriaxone	100	89	100	87		90	55	98	100	100	75	94	94	0	100	65
	Cefoxitin	100	100	95	90		100	100	64	78	75	100	81	75		100	40
	Cefotaxime	100	100	100	77		90	100	100	100	100	100	81	75	70	100	65
	Cefepime	100	100	100	100		100	100	100	100	100	88	100	100	70	100	90
Carbapenem	Imipenem	100	100	100	100		95	92	100	100	100	75	88	100	100	100	100
Fluoroquinolones	Ciprofloxacin	70	78	49	42		77	42	98	78	89	19	50	38	55	100	40
	Levofloxacin	100	95	60	87		42	90	45	78	96	56	38	31	40	100	50
Aminoglycosides	Gentamicin	100	100	100	87		100	100	78	89	96	75	50	81	10	10	0
	Kanamycin	nt	nt	nt	nt		nt	nt	nt	nt	nt	nt	nt	nt	0	0	0
Tetracycline	Tetracycline	68	38	49	67		82	50	64	45	85	56	56	56	0	10	0
	Tigecycline	nt	nt	nt	nt		nt	nt	nt	nt	nt	nt	nt	nt	0	0	0
Lincosamide	Clindamycin	89	78	89	87		77	90	78	91	64	81	56	75	nt	nt	nt
Macrolide	Erythromycin	89	67	68	77		58	82	64	78	71	44	50	44	0	0	40
	Colistin	nt	nt	nt	nt		nt	nt	nt	nt	nt	100	100	50	50	100	60
																	

All values are expressed as a percentage rounded up to the nearest whole number. nt=not tested, WWD=waste water derivatives, MSW=municipal solid waste, SF=shared fomites. Breakpoints of Comité de l’Antibiogramme de la Société Française de Microbiologie recommendations were used for tigecycline, gentamicin and tetracycline for *A. baumannii.*

### ESBL and MRSA detections

All samples were assessed for the presence of Gram-negative bacteria displaying resistance to at least penicillin, cephalosporin and/or fluoroquinolone antibiotics, with a focus on screening for ESBL production. ESBL production was confirmed in only seven *K *. *pneumoniae* isolates obtained from diapers, food wastes and restroom floors, five *A *. *baumannii* isolates from sludge and restroom floors, two *E. coli* isolates from sewage and one imipenem-resistant *P. aeruginosa* isolate also from sewage. Additionally, 10 of the isolated *S. aureus,* originating from restroom floors, door handles, polythene bags and sewage, were identified as MRSA, as detailed in Table 4.

**Table 4.** Screening of bacteria isolated from MSW, WWD and SF for MRSA and ESBL production

Type of resistance	Bacteria sp.	No. screened	No. positive (%)	Source
MRSA	* S. aureus *	72	10 (13.8)	Restroom floor Door handle Polythene bags Sewage
ESBL	* E. coli *	26	2 (7.69)	Sewage
	* K. pneumoniae *	34	7 (20.5)	Diaper Food remnant Restroom floor
	* P. aeruginosa *	12	1 (8.33)	Sewage
	* A. baumannii *	20	5 (25.0)	Sludge Restroom floor

### ARG detection in selected bacteria

Two ARGs, namely *bla*_*CMY-2*_ and *bla*_*NDM*_ were found within bacteria that were isolated from various sources, including sewage, sludge, plastics and restroom floors. Among these, bacteria isolated from sewage and restroom floors exhibited a higher prevalence of ARGs compared to other samples. Specifically, three *E. coli* strains isolated from sewage, sludge and restroom floors harboured the *bla*_*CMY-2*_, *bla*_*IMP*_ and *bla*_*NDM-1*_ genes, respectively. Notably, one ESBL-* K. pneumoniae* isolated from sewage co-harboured the *bla*_*KPC*_ and *bla*_*NDM-5*_ genes on its plasmid. ESBL-* P. aeruginosa* and non-ESBL producers carried the *bla*_*KPC*_ and *bla*_*CMY-2*_ genes, respectively (Table 5). The sequences of *bla*_*NDM*_ from two isolates showed a 99.82 % identity to the known NDM-5 gene sequence, whereas the sequence from the *E. coli* isolated from the restroom floor revealed the *bla*_*NDM-1*_ gene. The *bla*_*CMY-2*_ and *bla*_*KPC*_ genes exhibited nucleotide sequence identities ranging from 98 to 100 % with related strains in the database. Additionally, two *A. baumannii* strains isolated from sludge and a door handle harboured the *bla*_*KPC*_ and *bla*_*CMY-2*_ genes, respectively. When the assembled *bla*_*OXA-51-like*_ gene sequences from *A. baumannii* were subjected to Blastx database searchs, it was found that the isolates encode protein variants of *bla*_*OXA-66*_, known to be associated with clinical international clone *A. baumannii* lineages, whereas the isolate from the door handle belonged to the *bla*_*OXA-180*_ variant. Finally, six out of the 10 MRSA strains were found to carry the *mecA* genes.

**Table 5.** Detection of ARGs in randomly selected isolates from different sources

S/N	Isolate	Origin of isolate (no. screened)	Type and no. of ARGs detected						
			*bla_KPC_*	*bla_CMY-2_*		*bla_NDM_*	*bla_IMP_*	*MecA*	*bla_OXA-51_* variant
1	* E. coli *	Sewage (1)	–	1		–	–	na	na
		Sludge (1)	–	–		–	1	na	na
		Diapers (1)	–	–		–	–	na	na
		Restroom floor (1)	–	–		1	–	na	na
2	* K. pneumoniae *	Sewage (1)	1	–		1	–	na	na
		Sludge (1)	–	–		1	–	na	na
		Plastic (1)	–	1		–	–	na	na
		Restroom floor (1)	–	1		–	–	na	na
3	* P. aeruginosa *	Sewage (1)	1	–		–	–	na	na
		Sludge (1)	–	1		–	–	na	na
4	* A. baumannii *	Sludge (1)	–	1		–	–	na	*bla*_*OXA-66*_
		Restroom floor (1)	–	–		–	–	na	*bla*_*OXA-66*_
		Door handle (1)	1	–		–	–	na	*bla*_*OXA-180*_
5	* S. aureus *	Restroom floor (5)	na	na		na	na	3	na
		Door handle (3)	na	na	na	na	na	2	na
		Polythene bag (2)	na	na	na	na	na	1	na

na=not applicable; ARGs=antibiotic resistance genes.

### Efficacy of floor disinfection process routinely used in reducing burden of selected bacteria

A comparison was made between before and after a routine disinfection process of restroom floors, during which an unknown amount of disinfectant was used, to assess its effectiveness in removing ARB from the restroom floors. Nearly all the samples collected from the restroom floors in the morning before cleaning showed positive cultures for *S. aureus, E. coli, K. pneumoniae, P. aeruginosa* and *A. baumannii* throughout the study period. The floors exhibited higher contamination levels of *S. aureus, E. coli* and *K. pneumoniae* compared to *P. aeruginosa *. Specifically, *K. pneumoniae* and *S. aureus* were significantly more frequently recovered from locations near the toilet seat before cleaning when compared to *E. coli* and *P. aeruginosa* (*P*<0.05) (Table 6). There was a slight reduction in the cumulative number of bacteria on the surfaces observed 1 h after disinfection. Subsequent screening of the corridors after cleaning did not reveal the presence of *E. coli *, *K. pneumoniae* or *A. baumannii *, but *S. aureus* was still detected.

**Table 6.** Detection of bacteria on restroom floors and corridors for four consecutive days before and 1 h after cleaning and disinfection process

Time of sampling	Location of sampling	* S. aureus *				* E. coli *				* K. pneumoniae *				* P. aeruginosa *				*A.* *baumannii*			
		1	2	3	4	1	2	3	4	1	2	3	4	1	2	3	4	1	2	3	4
Before cleaning and disinfection	Near toilet seat	+	+	−	+	−	+	+	+	+	+	+	+	−	+	−	+	−	+	+	+
	Near door	+	−	+	+	+	+	+	+	+	−	+	+	+	+	−	−	−	−	−	−
	Corridor	+	+	+	−	+	−	+	−	−	+	−	−	−	−	+	−	−	−	−	−
	Total positive culture from sampling sites	3	2	2	2	2	2	3	2	2	2	2	2	1	2	1	1	0	2	1	0
1 h after cleaning and disinfection	Near toilet seat	+	+	−	+	−	+	−	+	+	+	+	−	−	−	+	+	−	+	+	+
	Near door	−	−	−	−	+	+	−	−	−	+	−	−	+	+	−	−	−	−	−	−
	Corridor	+	+	−	+	−	−	−	−	−	−	−	−	−	−	+	−	−	−	−	−
	Total positive culture	2	2	0	2	1	2	0	1	1	2	1	0	1	1	2	1	0	2	2	0

## Discussion

Despite a significant increase in antimicrobial resistance (AMR) and the spread of ARB such as *E. coli *, *S. aureus *, *K. pneumoniae *, *A. baumannii* and *P. aeruginosa* in hospital environments in low- to middle-income countries, as reported by WHO and Africa CDC [13, 14], there is limited information available regarding the types of ARB and their associated ARGs colonizing major components of MSW, sludge, sewage and shared fomites in Nigeria. Since sewage and wastewater have been shown to accurately reflect the population’s gut microbiota composition and antimicrobial use, it is crucial to conduct a comprehensive analysis of the bacterial communities present in community WWDs and MSW simultaneously.

All the samples from MSW, sewage, sludge and shared fomites that were screened yielded positive cultures, with a substantial proportion found in sludge and sewage. This finding is not surprising, as both sludge and sewage are rich in organic compounds that bacteria require for energy production. Sewage, which often contains faecal matter, harbours a slightly higher number of bacteria compared to sludge, which consists mainly of solids separated from wastewater originating from gutters, kitchens, toilets and nearby industries. Components of MSW, such as diapers and polythene bags used for sachet water, indiscriminately discarded in refuse dump sites, were found to harbour *E. coli, K. pneumoniae* and *S. aureus* at varying rates. Interestingly, none of the samples was contaminated with a single type of bacteria, suggesting a diverse environmental niche across the samples. While diapers are heavier and less likely to be blown away by the wind, polythene bags containing various bacteria could easily be transported through the air to other locations, including hospitals and school environments. Previous studies have also identified plastic debris [15], washroom floors [16], restroom surfaces [17], sewage soil [18], kitchen utensils [19], external surfaces, and alimentary tract of houseflies living on sewage [20] and food wastes [21] as surfaces and environmental reservoirs of bacterial pathogens such as *S. aureus, E. coli, Enterococcus faecalis, K. pneumoniae* and *A. baumannii *. Additionally, Butin *et al.* [22] isolated *Staphylococcus capitis* from several environmental sites inside the Neonatal Intensive Care Unit of a hospital.

The isolation of *K. pneumoniae* from all the samples is worrisome and indicates that this bacterium can readily establish itself in various environmental samples, similar to its persistence in clinical settings. Similarly, the recovery of *E. coli* and *K. pneumoniae* from nearly all components of MSW may suggest either easy transmission of these bacteria among MSW components or high faecal contamination in dump sites from which each component of MSW is collected. The frequent recovery of *A. baumannii* and *K. pneumoniae* from restroom floors and other fomites may be attributed to their versatile genetic machinery, which allows them to tolerate harsh environments, form biofilms and adhere to surfaces. Overall, the high volume of waste and dirt accumulated on restroom floors, shared by many students and ranging from tissue papers used for cleaning to sputum and urine discharged on the floors, may be a contributory factor to the persistent isolation of gastrointestinal bacteria [23].

The high resistance of bacteria isolated from all environmental wastes and shared fomites to penicillin is consistent with the high resistance often observed among clinical and veterinary isolates [24, 25]. Some bacteria (*P. aeruginosa, A. baumannii* and *S. aureus *) isolated from sewage and sludge have also developed resistance to fluoroquinolones, tetracyclines and colistin. Similar observations in animals have been reported by Egbule and Yusuf [5], where isolates from poultry and cattle dung in environments have developed significant resistance to common antibiotics. The majority of bacteria isolated are susceptible to other antibiotics, despite exhibiting some level of resistance to the same antibiotics among clinical samples. In line with this finding, resistance to penicillin and fluoroquinolones by bacteria isolated from landfills and laboratories has been reported to have increased in studies conducted in Ghana and Zimbabwe [26, 27]. Kanamycin, gentamicin and ampicillin resistance by *A. baumannii* have been widely reported among clinical isolates [28] as well as intrinsic resistance to chloramphenicol [29].

The production of ESBL by *E. coli* and *K. pneumoniae* isolated from some components of MSW, WWDs and shared fomites aligns with the high-level recovery of ESBL-* E. coli* from clinical samples in the same environment [9, 30]. This could indicate the presence of hospital waste in the environments sampled in this study. However, the detection of ESBL-* K. pneumoniae* in sewage and ESBL-* P. aeruginosa* in diapers and food waste could suggest contamination from households.

The presence of *bla*_*NDM-1*_ and *bla*_*KPC*_ in two different *K. pneumoniae* isolates and *bla*_*IMP*_ in one non-ESBL *E. coli* isolated from sewage and sludge, respectively, is of public health concern and corresponds to the findings of previous studies in different regions of Nigeria where *bla*_*NDM*_ and *bla*_*KPC*_ have been reported in clinical samples [31, 32]. It is worth noting that *A. baumannii* genotypically harbouring *bla*_*OXA-51-like*_ and *bla*_*CMY-2*_ genes is phenotypically susceptible to imipenem and cephalosporins, probably due to the lack of insertion sequence ISAba1 adjacent to *bla*_*OXA-51-like*_, which serves as a promoter for carbapenem resistance expression.

**Fig. 2.**
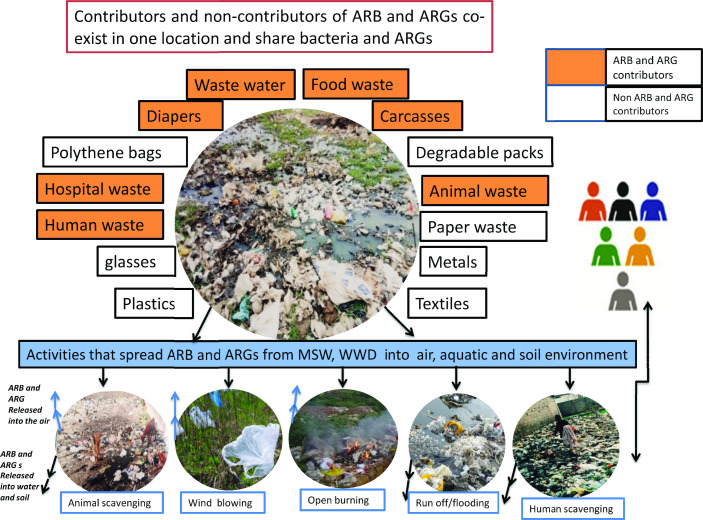
The big picture of a how municipal solid wastes and waste waters indiscriminately discharged into the environment in Nigeria generate and transmit ARB and ARGs. The diagram explains how different human activities are being carried out in the communities (such as burning wastes, scavenging by humans and animals). Airborne bacterial pathogens are released during burning, wind blowing, and loading and unloading of garbage by waste dischargers, and scavenging humans and animals and could lead to the spread of ARB to the nearby human population and fomites.

Although the source of these bacteria is not clear at present, it could be linked to close interactions between humans, animals and waste, facilitating the exchange of pathogens through the activities of waste scavengers, open defecators in waste disposal sites, and runoff from disposal sites to houses and vice versa (see Fig. 2). Previous reports have indicated the presence of resistant genes in river water in Vietnam [33], seepage and tap water in India [34], and *bla*_*IMP-1*_ in Tunisian rivers [35]. Another possible reason is that freely moving wild animals have access to waste disposal sites, and ARB shed by these animals may contaminate the soil and other waste dumped on it. Similarly, wild animals can come into close contact with sewage and sludge, leading to the widespread dissemination of ARB and ARGs originally found in hospitals to the environment [36].

Finally, the partial failure of the disinfection process to completely remove bacteria, specifically ARB, may be explained by a decreased susceptibility of the bacteria to the disinfectants used during cleaning. Shorter contact times, inappropriate concentrations and technical difficulties in reaching hidden areas of the restroom may have limited the effectiveness of the disinfectants in exerting their bactericidal effects, thus favouring the persistence of bacteria.

## Conclusion

The present study has established that municipal solid waste indiscriminately discharged in some urban cities in Nigeria, as well as sewage, sludge and frequently shared fomites, often harbour ARB such as *S. aureus, E. coli, K. pneumoniae, P. aeruginosa* and *A. baumannii *. These bacteria display resistance to penicillin, tetracycline and fluoroquinolones. Furthermore, ESBL-producing strains of *K. pneumoniae, E. coli, P. aeruginosa* and *A. baumannii *, as well as other non-ESBL producers, carry one or more ARGs, including *bla*_*KPC*_, *bla*_*NDM-1*_, *bla*_*CMY-2*_ and *bla*_*IMP*_, which are commonly found in hospital-acquired strains. Notably, the disinfection process employed in the university did not effectively reduce the levels of ARB. Therefore, we advocate for an informed One Health strategy to address the role of the environment in the emergence and spread of AMR in Nigerian urban communities.
